# Survival, healthcare resource use and costs among stage IV ER + breast cancer patients not receiving HER2 targeted therapy: a retrospective analysis of linked SEER-Medicare data

**DOI:** 10.1186/1472-6963-14-298

**Published:** 2014-07-09

**Authors:** Kathleen Lang, Huan Huang, Medha Sasane, Victoria Federico Paly, Yanni Hao, Joseph Menzin

**Affiliations:** 1 Boston Health Economics, Inc, Waltham, MA, USA; 2 Novartis Pharmaceuticals Corporation, East Hanover, NJ, USA

## Abstract

**Background:**

Few studies have evaluated survival, treatment, resource use, and costs among women with stage IV ER + breast cancer (BC) who did not receive HER2 targeted therapy.

**Methods:**

Using linked Surveillance, Epidemiology, and End Results (SEER) and Medicare data from 2006-2009, women aged 66+ years with an incident diagnosis of stage IV ER + BC (index date) in 2007 and no HER2 targeted therapy were identified. A comparison cohort without cancer was created from the SEER 5% Medicare sample and matched 1:1 to the study cohort based on age, sex, and race. All patients had continuous enrollment for a 12-month baseline period prior to index and were followed until the end of the study window, disenrollment, or death, whichever came first. Resource utilization and costs (by place of service, reported per patient per month, PPPM) were compared across cohorts. Treatment patterns including receipt of surgery, radiation, chemotherapy, aromatase inhibitors (AI), and non-AI hormonal therapy were evaluated for study cohort patients with at least 2 months of follow-up. Kaplan-Meier survival analysis was also conducted.

**Results:**

325 women with stage IV ER + BC without HER2 targeted therapy were identified and matched to 325 women without cancer. Mean age was 77 years for both cohorts, with average follow-up of 18 months for study patients and 26 months for comparison patients. Compared to the comparison cohort, study patients had significantly higher mortality (60.3% versus 31.1%, *P* < 0.001), shorter survival (survival at 36 months 28% vs. 62%) and higher resource utilization across all settings except for oral prescription drugs. Total PPPM healthcare costs were also significantly higher among study patients ($7,271 vs. $1,778, *P* < 0.001). Approximately 57% of study patients with 2+ months of follow-up received chemotherapy and over 62% received an AI during follow-up. Within 4 months of cancer diagnosis, surgery and radiation were received by 39% and 32% of study patients, respectively.

**Conclusions:**

We found significant excess clinical and economic burden among women with stage IV ER + breast cancer who did not receive HER2 targeted therapy. Future studies with more precise and recent data are warranted to confirm and extend these results.

## Background

Breast cancer is the most common invasive cancer in women and the second leading cause of death from cancer among women in the United States (US) [1]. Approximately 1 in 8 women will be diagnosed with breast cancer during her lifetime, and within the US in 2013 there will be 232,340 newly diagnosed cases and 39,620 deaths associated with the disease [2]. The majority of these deaths result from recurrent or metastatic disease, which occurs in 6% of newly diagnosed patients and approximately 30% of those previously diagnosed with an earlier stage [3].

Treatment options for metastatic breast cancer include systemic therapies (chemotherapy, hormone therapy), surgery, and/or radiation [4]. Systemic therapies are the recommended primary treatment option for patients with metastatic breast cancer, while surgery is generally considered, if appropriate, after initial systemic therapy [4]. Radiation therapy may be considered as an alternative to surgery or as palliative therapy [4].

Among other factors, tumor biology and clinical features influence therapeutic strategy. The National Comprehensive Cancer Network (NCCN) guidelines recommend all patients newly diagnosed with breast cancer be tested for hormone receptor status via expression of estrogen and/or progesterone receptors (ER/PR) and human epidermal growth factor receptor-2 (HER2) [4]. Endocrine therapy is the preferred treatment for patients with stage III or IV hormone-receptor-positive (HR +, which includes ER and/or PR positive) breast cancer, but chemotherapy may also be recommended for cases with rapidly progressive disease [4,5]. Recommended endocrine therapies for HR + patients include tamoxifen, fulvestrant, megestrol, and aromatase inhibitors, such as anastrazole, letrozole or exemestane [4]. In addition, targeted therapies such as everolimus plus exemestate (Afinitor, Novartis Pharmaceuticals, Basel) have recently been approved or are in further clinical development for HR + advanced breast cancer [6].

Few population-based studies of survival among women with advanced HR + breast cancer exist. A recently published study of SEER registry data by Johnson and colleagues estimated 5-year survival of distant breast cancer to be 31% [7]; however, Johnson did not report survival by hormone receptor status. Jung and colleagues reported median survival of approximately 45 months among women with either ER or PR positive metastatic BC in a single large urban practice [8]. Furthermore, there are limited published studies on resource use and costs in this population. A recent systematic review of the literature on the burden of ER + advanced breast cancer found only one study on the economic burden of ER + patients which was focused on the impact of recurrence [9]. Given that approximately 80% of ER + patients would not be indicated to receive HER2 targeted therapy [10], we have focused our study on those ER + patients who did not receive a HER2 targeted therapy.

We conducted a retrospective database analysis of survival, treatment patterns, healthcare resource use, and costs using the Surveillance and Epidemiology End Results database linked to Medicare claims (SEER-Medicare). We identified women diagnosed with stage IV ER + breast cancer who received immunohistochemistry or fluorescence in situ hybridization (FISH) testing and did not receive a HER2 targeted agent (trastuzumab or lapatinib) and compared them to a matched comparison cohort of women without cancer. This primary population was chosen as women with ER + and HER2 - breast cancer represent the largest patient population among all BC patients.

## Methods

### Data source

The linked SEER-Medicare database is a collaborative effort of the National Cancer Institute (NCI), the SEER registries, and the Centers for Medicare and Medicaid Services (CMS). SEER is an epidemiologic surveillance system consisting of population-based tumor registries designed to track cancer incidence and survival in the US. The registries routinely collect information on newly diagnosed cancer patients in geographically defined areas that represent approximately 25% of the US population. The registries ascertain all newly diagnosed cancer cases from multiple reporting sources such as hospitals, outpatient clinics, laboratories, private clinics, nursing homes, hospice, autopsy reports, and death certificates. The linked SEER-Medicare data files are not publicly available; investigators and researchers must obtain approval from the NCI for specific research objectives in order to obtain the data. Approval is granted at the discretion of the NCI to ensure confidentiality and protection of the patients and providers in SEER registries. Such approval and subsequent access to the data for this study were granted by the NCI following submission of a formal data request outlining the research objectives. The National Institutes of Health's Office of Human Subjects Research has determined that analyses using SEER-Medicare data are exempt from requiring further IRB review and approval.

The database used in this study included breast cancer cases diagnosed in 2007 with Medicare claims from 2006 through 2009. In addition, data for a 5% Medicare sample were used to create a comparison cohort. The database includes a SEER file of patients diagnosed with cancer within the geographic areas covered by SEER registries (Patient Entitlement and Diagnosis Summary [PEDSF] file), as well as Medicare claims covering the period up to two years after the last year of available SEER data. The Medicare administrative claims files include individual claims for inpatient and skilled nursing facility (SNF) hospitalizations (Medicare Provider Analysis and Review [MEDPAR] file), outpatient hospital visits and miscellaneous ambulatory services (Outpatient file), home health agency services (HHA file), hospice services (Hospice file), carrier claims (NCH file, formerly the Physician/Supplier file), durable medical equipment (DME file), and Part D prescription drug claims (PDE file). Part D prescription data were only available for 2007-2009.

### Patient selection and follow-up

Two cohorts were selected for this study: a cohort of stage IV ER + breast cancer patients who received an immunohistochemistry or FISH test and did not receive a HER2 targeted agent (study cohort), and a matched comparison cohort of women who did not have cancer at the start of follow-up (comparison cohort). All patients in both cohorts were required to meet the following criteria:

● 66 years or older at index date (to allow 1 year baseline period);

● Entitlement for both Part A AND Part B Medicare benefits at all points during the study period (including a 12-month baseline period prior to index date);

● Not enrolled in an HMO during any study month;

● Medicare entitlement not based on end stage renal disease (ESRD) or disability; and

● No claims after date of death

Female patients with an incident diagnosis of stage IV breast cancer (SEER site recode 46, AJCC stages 'IV', 'IVNOS', 'IVA', 'IVB', or 'IVC') between 1/1/2007 and 12/31/2007 were identified for possible inclusion in the study cohort, with the date of cancer diagnosis serving as the index date. This cohort was then limited to women who met the following additional criteria:

● Breast cancer was identified as the patient’s primary cancer using SEER variables indicating the order of incident cancer diagnoses for a given patient (i.e., first cancer site was the primary cancer).

● Breast cancer was ER +, identified using the SEER breast cancer site-specific factor variable for an estrogen receptor assay. Patients were classified as ER + if the ER assay was positive/elevated

● Received an immunohistochemistry or fluorescence in situ hybridization (FISH) test during or following the month of breast cancer diagnosis. Tests were identified if any of the following CPT codes were noted on a claim: 88342, 88360, 88361, 88365, 88271, 88274, 88291, 88367, 88368, 83950

● Did not receive a HER2 targeted agent (i.e., trastuzumab (Herceptin®, Roche), lapatinib (Tykerb, GSK) any time after the test mentioned above

● No (prior) history of any other (non-breast) cancer diagnosis before breast cancer diagnosis

● Diagnosis of breast cancer was not at time of death or autopsy

To create the comparison cohort, one randomly selected patient of identical age, sex and race was matched to each study cohort patient and assigned the same index date (to follow them over the same time period). Comparison patients were selected from the Medicare enrollment files using a 5% sample of Medicare beneficiaries residing in SEER areas who had not been reported to any of the SEER registries as having any cancer prior to their index date. These patients were not required to have used services in order to be selected for inclusion, and were allowed to develop cancers other than breast cancer after their index date. All patients were followed until the end of the study window (12/31/2009), disenrollment, or death, whichever came first.

### Study measures

Baseline demographic and clinical characteristics were assessed over a 12-month period preceding the index date. These included age, race, region, urban/rural area, Charlson comorbidities and score [11,12], presence of other comorbidities commonly present among breast cancer populations such as osteoporosis and fractures (identified based on the presence of any of the following ICD-9 diagnosis codes: 733.0x, 805.xx, 807.0x-807.4x, 808.xx, 809.xx, 813.xx-814.xx, 820.xx-821.xx, 733.1x) and depression or anxiety (identified based on the presence of any of the following ICD-9 diagnosis codes: 296.2x, 296.3x, 300.4, 309.0x, 309.1x, 311.xx), and progesterone receptor (PR) status (identified using the SEER breast cancer site-specific factor variable for a PR assay). Mortality rates and time from index date to death were also evaluated, with Kaplan-Meier survival analysis conducted for overall survival.

Among patients in the study cohort, we evaluated the percent undergoing surgery related to the primary cancer site or radiation within 4 months of initial breast cancer diagnosis using SEER-created summary fields. We also evaluated the percent of patients prescribed chemotherapy, aromatase inhibitors (AI), and non-aromatase inhibitor hormonal therapy (non-AI). AIs included anastrazole, letrozole, and exemestane. Non-AIs included fulvestrant, tamoxifen, toremifene, and megestrol. Receipt of each treatment (i.e., chemotherapy, AIs, non-AIs) was identified based on the presence of the corresponding NDC codes or HCPCS codes. These treatment patterns were only evaluated among patients with at least 2 months of follow-up to allow for treatment observation. Patients who received more than one type of treatment during the follow-up period were considered to have received combination regimens.

Resource utilization during the follow-up period was assessed using the following measures: percent with at least one hospitalization, number of hospital admissions (defined as number of unique records in the inpatient claims file as each record represented a unique hospitalization), total hospital days per patient (defined as the sum of hospital days for all hospitalizations for a given patient), percent with office/clinic visits and number of visits (defined as number of unique days with such visits), percent with physician/provider claims and number of visits (defined as number of unique days with such claims), percent receiving prescriptions for oral drugs, percent with home health care use, percent with skilled nursing facility (SNF) use, percent with hospice use, and percent with durable medical equipment (DME) use. The setting of resource use was based on the source file of the encounter, as SEER-Medicare provides separate claims files for inpatient, outpatient office/clinic, physician/provider, Part D pharmacy, home health care, SNF, hospice, and DME encounters. Total healthcare costs during the follow-up period were estimated by summing all Medicare payments and patient copayments and deductibles across all settings. Total costs for each resource use setting were also evaluated.

### Data analysis

Descriptive analyses of all study measures were performed. Binary variables were summarized using percentages and continuous variables were summarized using means, standard deviations (SD), and medians. Analyses of resource use and costs were conducted on a per-patient-per-month (PPPM) basis to allow for variable follow-up lengths. Statistical testing between the two cohorts was conducted for mortality rates, healthcare costs, and overall survival. Continuous variables were tested using the nonparametric Wilcoxon test, proportions were tested using the Fisher’s exact test, and survival was tested using the Log-Rank test. All cost measures were adjusted to 2011 US dollars using the medical care component of the consumer price index. All analyses were conducted using SAS software (Version 9.3, SAS Institute, Cary, NC).

## Results

### Patient selection and baseline demographic characteristics

**Figure 1:**
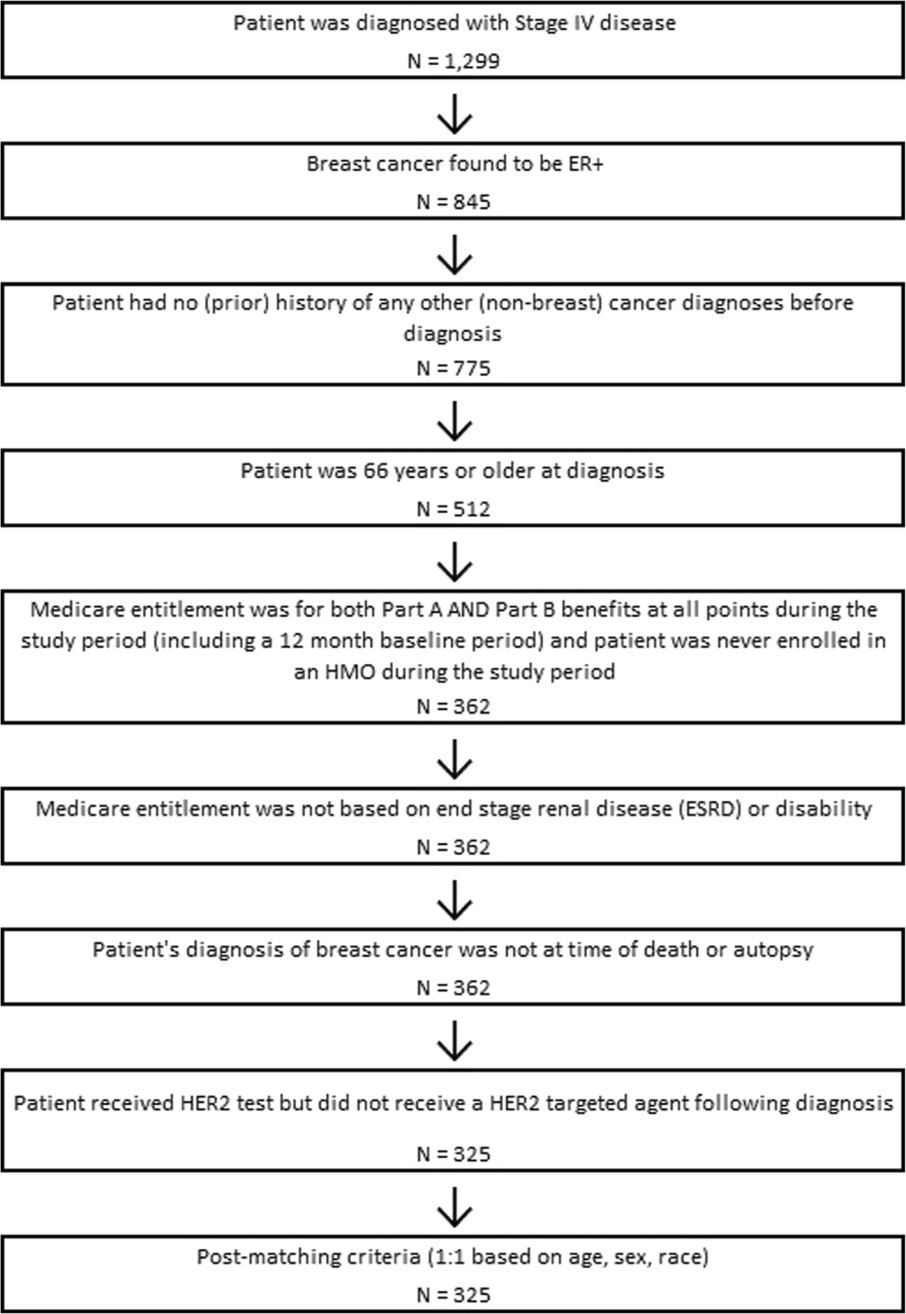
Patient selection.

A total of 325 women meeting all eligibility criteria were identified as having an incident diagnosis of stage IV ER + breast cancer and no HER2 targeted therapies following diagnosis. Figure 1 presents the full patient selection results. Among the 325 comparison patients, 7 patients (2.2%) developed a non-breast cancer during the follow-up period, and the remaining 318 patients did not have any cancer diagnoses during follow-up.

The mean age for both cohorts was 77 years, with 37% of patients being 80 years or older. The majority of patients (85.5%) were white, followed by black (10.2%) and Asian (2.2%). Over 99% of study patients and approximately 97% of comparison patients were from an urban setting. Compared to comparison patients, there were more study patients in the Northeast (30.8% vs. 19.1%) and fewer from the West (34.8% vs. 42.5%). Average follow-up was shorter for study patients (18.1 vs. 25.8 months) compared to comparison patients (see Table 1), which included the period from index date to the end of the study window (12/31/2009), disenrollment, or death, whichever came first.

**Table 1:** Sociodemographic and clinical characteristics and duration of follow-up for Medicare-eligible stage IV ER + breast cancer patients who did not receive HER2 targeted agents and matched comparison patients

**Characteristic**	**Study cohort**	**Comparison cohort**	*P*-value***
N	325	325	
Age (years)*			
Mean ± SD	77.18 ± 7.19	77.26 ± 7.75	0.813
Median	77.00	76.00	
Race (%)*			1.000
White	85.5%	85.5%	
Black	10.2%	10.2%	
Hispanic	0.6%	0.6%	
Asian	2.2%	2.2%	
Other	1.5%	1.5%	
Urban (%)	99.1%	96.6%	0.055
Geographic region (%)			0.005
Northeast	30.8%	19.1%	
Midwest	13.8%	12.9%	
West	34.8%	42.5%	
South	20.6%	25.2%	
Duration of follow-up (months)			
Mean ± SD	18.12 ± 11.91	25.82 ± 8.94	< 0.001
Median	20.00	28.00	
Charlson score**			
Mean ± SD	1.12 ± 1.58	1.31 ± 1.71	0.072
Median	1.00	1.00	
Charlson comorbidities (%)			
Diabetes without chronic complications	25.8%	26.5%	0.929
Chronic pulmonary disease	23.1%	20.3%	0.447
Congestive heart failure	14.2%	12.0%	0.485
Cerebrovascular disease	10.8%	16.3%	0.051
Diabetes with chronic complications	7.7%	8.0%	1.000
Renal disease	6.2%	7.1%	0.753
Peripheral vascular disease	5.8%	8.9%	0.177
Rheumatologic disease	4.3%	5.2%	0.713
Myocardial infarction	3.7%	4.3%	0.842
Dementia	2.2%	8.3%	< 0.001
Peptic ulcer disease	0.9%	2.2%	0.340
Hemiplegia or paraplegia	0.3%	1.8%	0.123
Moderate or severe liver disease	0.3%	0.3%	1.000
AIDS	0.0%	0.0%	< 0.001
Mild liver disease	0.0%	0.6%	0.499
Other comorbidities (%)			
Depression/anxiety	9.2%	17.8%	0.002
Osteoporosis/Fractures	14.5%	25.8%	< 0.001
PR status			
PR+	78.0%	n/a	
PR-	22.0%	n/a	

*Matching criteria.

**Charlson score excludes any primary malignancy and metastatic solid tumor.

***Proportions were compared using the Fisher's exact test while continuous measures were compared using the nonparametric Wilcoxon test.

### Clinical characteristics

Baseline Charlson scores (excluding any primary malignancy and metastatic solid tumor) were relatively similar between the two cohorts (1.1 ± 1.6 [mean ± SD] among study patients and 1.3 ± 1.7 among comparison patients). Diabetes, chronic pulmonary disease, cerebrovascular disease and congestive heart failure were common comorbidities in both cohorts. Over 9% of the study cohort and almost 18% of the comparison cohort had depression/anxiety during the baseline period (*P* = 0.002). Similarly, osteoporosis or osteoporosis-related fracture prevalence was significantly lower in the baseline period for study patients (14.5% vs. 25.8%, *P* < 0.001). Over three-quarters of study patients were PR + (78%) (see Table 1).

### Treatment patterns

Among the 285 (87.7%) study patients with at least 2 months of follow-up (to allow for treatment analysis), 38.6% received surgery and 31.9% received radiation within 4 months of their initial breast cancer diagnosis. Approximately three-quarters (74.4%) of these patients were prescribed some type of cancer medication (see Table 2). More than half of patients (57.1%) were prescribed chemotherapy at some point after their diagnosis (15.1% chemotherapy only, 15.1% chemotherapy with a non-AI, 15.1% chemotherapy with both an AI and non-AI, and 11.8% chemotherapy with an AI). Receipt of both an AI and a non-AI during follow-up was also common (10.4%), as was anastrazole monotherapy (11.8%) and letrozole monotherapy (9.4%). Over 62% of patients were prescribed an AI at some point during follow-up. Among those who received any medication, the mean (±SD) duration of treatment was 12.9 months (±9.1). The mean durations for AI, non-AI and chemotherapy among those who received them were 11.0 (±8.1), 5.6 (±5.4), and 8.5 (±7.2) months, respectively.

**Table 2:** Treatment patterns among Medicare-eligible Stage IV ER + breast cancer patients who did not receive HER2 targeted agents

**Characteristic**	**Study cohort**
With > 2 months of follow-up to allow for treatment	
N	285
%	87.7%
Receiving surgery within 4 months of diagnosis	
N	110
%	38.60%
Receiving radiation within 4 months of diagnosis	
N	91
%	31.93%
Receiving any medication	
N	212
%	74.4%
Type of treatment received (at any time post-index)	
Chemotherapy alone	15.1%
Aromatase Inhibitors (AI) therapy	
Anastrazole monotherapy	11.8%
Letrozole monotherapy	9.4%
Exmenestane monotherapy	1.4%
Multiple AI therapies	2.4%
Non-AI therapy	
Fulvestrant monotherapy	4.2%
Tamoxifen monotherapy	1.9%
Toremifene monotherapy	0.0%
Megestrol monotherapy	0.9%
Multiple non-AI therapies	0.5%
Chemotherapy combined with AI therapy	11.8%
Chemotherapy combined with non-AI therapy	15.1%
AI therapy combined with non-AI therapy	10.4%
Chemotherapy combined with AI and non-AI therapy	15.1%
Duration of any treatment (months)	
Mean ± SD	12.9 ± 9.1
Median	11.0
Duration of AI treatment (months), among those with any AI use	
Mean ± SD	11.0 ± 8.1
Median	9.5
Duration of non-AI treatment (months), among those with any non-AI use	
Mean ± SD	5.6 ± 5.4
Median	4.0
Duration of chemo treatment (months), among those with any chemo use	
Mean ± SD	8.5 ± 7.2
Median	6.0

### Survival

**Figure 2:**
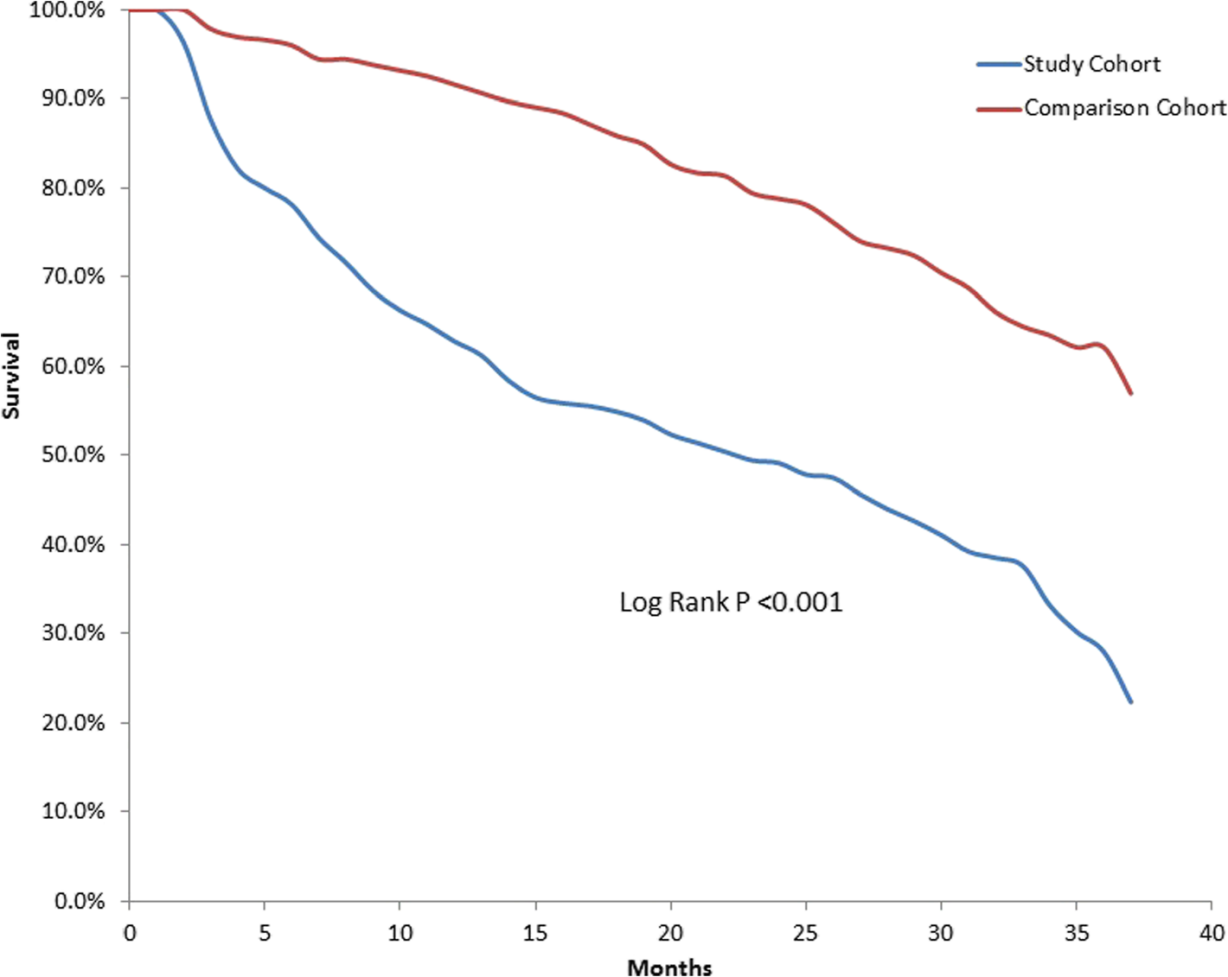
**Kaplan-Meier survival analysis for Medicare-eligible stage IV ER + breast cancer patients who did not receive HER2 targeted agents and matched comparison patients.** Note: Median survival for the study cohort was 23 months and it was not reached for the comparison cohort during the study period.

Almost twice as many study patients died during the evaluation period compared to comparison patients (60.3% vs. 31.1%, *P* < 0.001). The median time to death among those who died was 8 months for study patients and 19 months for comparison patients (*P* < 0.001). In Kaplan-Meier survival analysis, median survival for study patients was 23 months and was longer than the follow-up window for comparison patients (see Figure 2).

### Resource use and costs

Study patients had significantly higher resource utilization levels across most resource settings, including inpatient utilization (83.1% with hospitalization vs. 47.4%, *P* < 0.001), use of physician/provider services (100.0% vs. 98.2%, *P* = 0.031), SNF care (33.2% vs. 17.8%, *P* < 0.001), home health care (47.4% vs. 21.2%, *P* < 0.001), hospice use (34.2% vs. 7.7%, *P* < 0.001), and use of DME (60.0% vs. 45.5%, *P* < 0.001; see Table 3). While the percent of patients with outpatient office/clinic visits was not different (90.2% vs. 84.9%, *P* = 0.057) the mean PPPM number of visits was significantly higher among study patients (0.9 vs. 0.4, *P* < 0.001). The percent of patients receiving any prescription drugs (covered by Part D) was not different between the two cohorts (58.5% vs. 60.0%, *P* = 0.750).

**Table 3:** Resource utilization for Medicare-eligible stage IV ER + breast cancer patients who did not receive HER2 targeted agents and matched comparison patients

**Characteristic**	**Study cohort**	**Comparison cohort**	*P*-value*
N	325	325	
Percent hospitalized	83.1%	47.4%	< 0.001
Hospital admissions (PPPM)			
Mean ± SD	0.2 ± 0.3	0.1 ± 0.1	< 0.001
Median	0.1	0.0	
Hospital days (PPPM)			
Mean ± SD	2.1 ± 3.2	0.4 ± 1.0	< 0.001
Median	0.7	0.0	
Outpatient			
Office/clinic visits			
Percent with outpatient office/clinic visits	90.2%	84.9%	0.057
Outpatient office/clinic visits (PPPM)			
Mean ± SD	0.9 ± 0.9	0.4 ± 0.6	< 0.001
Median	0.8	0.2	
Physician/provider claims			
Percent with physician/provider claims	100.0%	98.2%	0.031
Physician/provider visits (PPPM)			
Mean ± SD	4.5 ± 3.2	1.8 ± 1.6	< 0.001
Median	3.5	1.4	
Percent receiving prescription drugs**	58.5%	60.0%	0.750
Percent receiving SNF care	33.2%	17.8%	< 0.001
Percent receiving home health care	47.4%	21.2%	< 0.001
Percent receiving hospice care	34.2%	7.7%	< 0.001
Percent with DME claims	60.0%	45.5%	< 0.001

*Proportions were compared using the Fisher's exact test while continuous measures were compared using the nonparametric Wilcoxon test.

**Covered by Part D.

DME: Durable Medical Equipment; PPPM: per-patient-per-month; SNF: Skilled Nursing Facility.

**Figure 3:**
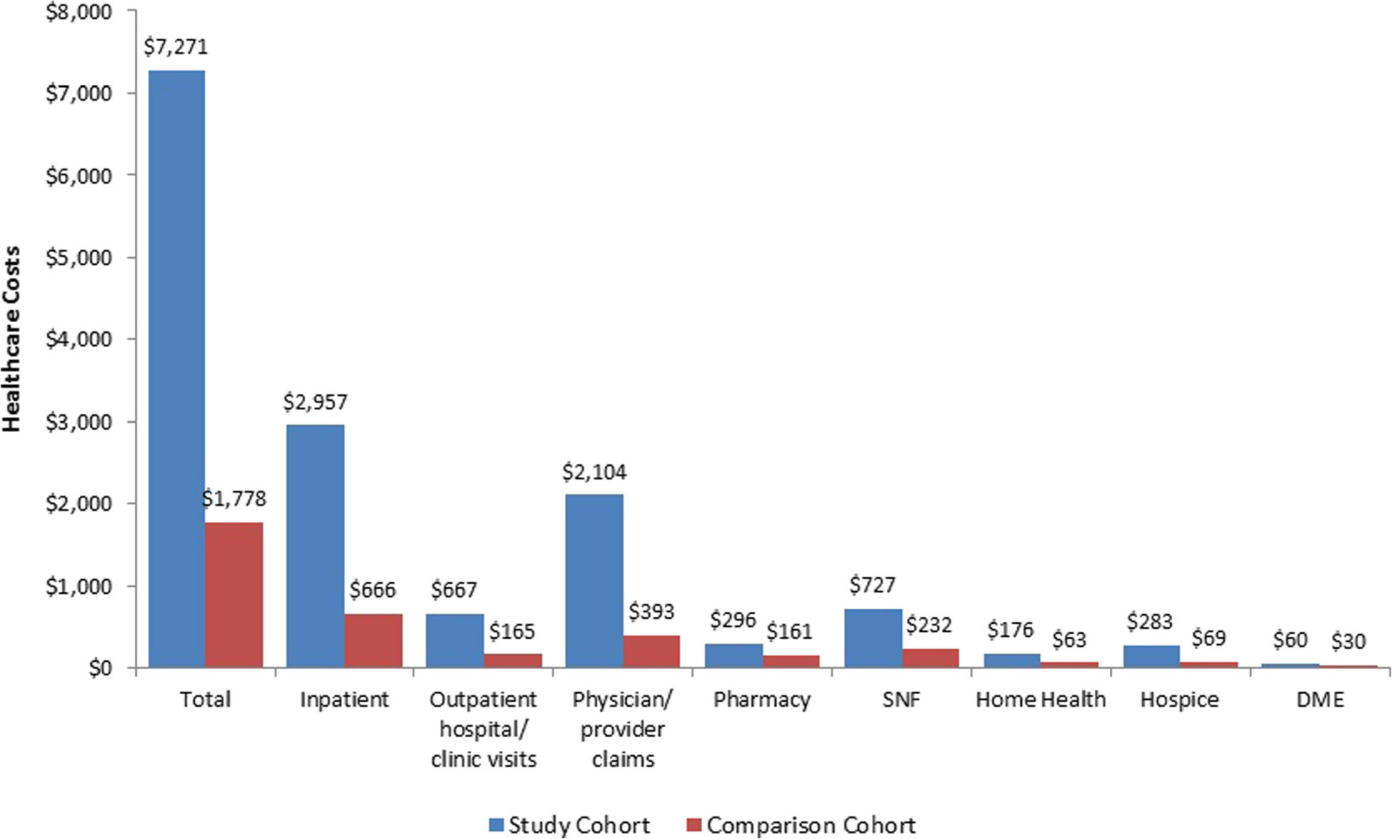
**Per-patient-per-month health care costs for Medicare-eligible stage IV ER + breast cancer patients who did not receive HER2 targeted agents and matched comparison patients.** Note: All differences between the two cohorts were significant at P <0.001, with the exception of Pharmacy which was P = 0.082. Statistical significance was tested using the nonparametric Wilcoxon test.

Total PPPM healthcare costs were over four times higher among study patients $7,271 vs. $1,778, *P* < 0.001). The major cost drivers were inpatient care ($2,957 vs. $666, *P* < 0.001) and physician/provider services ($2,104 vs. $393, *P* < 0.001). Similar to resource utilization, study patients had significantly higher costs across all resource settings except pharmacy costs for oral drugs (see Figure 3).

## Discussion

### Summary

This study compared a cohort of women diagnosed with incident stage IV ER + breast cancer who received immunohistochemistry or FISH test and did not receive a HER2 targeted agent to an age, sex and race matched cohort of patients without cancer. The mortality rate in the study cohort was almost double that in the comparison cohort (60% versus 31%). Not surprisingly, study patients also had significantly higher rates of resource utilization and healthcare costs on a PPPM basis. While all study patients were initially diagnosed with metastatic breast cancer, almost one-third of these women underwent surgery or radiation within four months of diagnosis. Additionally, approximately 57% of study patients received some type of chemotherapy and over 62% received an AI during the follow-up period.

### Comparison to literature

There are limited published studies evaluating burden of disease in women with stage IV ER + breast cancer. Papers that do report survival by ER status seem to report a wide range of estimates. A recent systematic literature review by Boswell and colleagues [9] identified only one study during 2000-2011 that evaluated burden of disease based on ER status [13]. This study by Stokes *et al.* found that elderly women diagnosed with Stage I-III ER + breast cancer who, using a claims-based algorithm, appeared to have a distant recurrence survived a median of 9 months following recurrence [13]. This is much lower than our median survival estimate of 23 months, likely due to the fact that the population in the Stokes *et al.* paper was diagnosed almost 20 years ago and there were fewer therapies available (e.g., almost no hormonal therapy available). In addition, Stokes *et al.* consisted of patients diagnosed at earlier stages than our population with incident ER + metastatic cancer. Jung and colleagues examined survival among metastatic breast cancer patients of all ages who were diagnosed between 1999 and 2008, and found that those with ER +/PR + status had a median survival of 45 months [8]. This was much longer than we observed (23 months among all ER + patients and 26 months among ER +/PR +), as would be expected given the younger patient population studied (median age of 55 vs. 77 years in our study). Rao *et al.* examined Medicare-eligible women with metastatic breast cancer as well and found a similar mortality rate as in our study (68% in the Rao *et al.* and 60% in our study); however, median survival was shorter (15 months vs. 23 months in our study) [14]. This difference may be expected as their study included all metastatic breast cancer patients and ours was restricted to those that were ER + and not receiving a HER2 targeted agent. It has been shown that patients with ER + breast cancer have better survival rates than other sub-types of breast cancer [10].

We found a significant excess cost burden among stage IV ER + breast cancer patients not treated with HER2 targeted therapy. Rao and colleagues also compared Medicare eligible women with metastatic breast cancer to non-cancer controls, finding significantly higher costs among those with breast cancer [14]. When adjusted to 2011 USD and standardized to a PPPM rate, their estimate of total healthcare costs for the cancer cohort was $3,511, which is about half of our PPPM estimate of $7,271 in health care costs for study patients. As described above, a similar proportion of the population died during the evaluation period, but the median time of survival was shorter and there were twice as many hospitalizations PPPM observed in the follow-up period among our study patients (0.10 vs.0 .24 hospitalizations in our study). This disparity may also be related to difference in the population evaluated (all metastatic breast cancer in Rao vs. stage IV ER + and not receiving trastuzumab or lapatinib in our study).

Despite the fact that surgery of the primary site is not regularly recommended in patients with primary metastatic cancer [4,15], we observed a relatively high rate of surgery in the first four months after diagnosis. This is not unexpected given several recent studies that have found that resection of the primary tumor in metastatic patients may improve survival [15-18].

### Limitations

This study is subject to the limitations of retrospective claims-based analyses, such as coding errors and incomplete data [19]. The SEER-Medicare database is not representative of all patients in the United States and does not capture those with other forms of health insurance (e.g., managed care, private). Additionally, only women over the age of 65 were evaluated. Therefore, this population may not be representative of the entire stage IV ER + breast cancer population. Medicare claims were only available through 2009 and may not capture all relevant healthcare costs. In addition, only treatments available up to 2009 were captured in the analysis, so recently approved therapies, such as everolimus, were not part of this analysis. Furthermore, continuous enrollment in Part D was not a requirement for study inclusion and therefore we may not have complete pharmacy claims for all patients, limiting the treatment data available. The sequence of treatment (e.g., first line/second line treatment) was not evaluated in this study as it was not a primary research objective, however, future studies evaluating lines of therapy would be beneficial in this population.

An additional limitation of this study is it is not clear why patients in the study cohort did not receive HER2 targeted therapy. This may be related to the patient’s HER2 status, or the presence of a contraindication for HER2 targeted therapy. In the years of data available, HER2 status was not collected by the SEER registries, and therefore we were unable to analyze the reason patients did not receive HER2 targeted therapy. Fortunately, SEER began collecting data on HER2 status in 2010.

Because the HER2 targeted agent lapatinib is an oral drug and Part D pharmacy claims were only available from 2007 forward, we were unable to include cases from previous years, limiting our sample size. This study only included women with an incident diagnosis of stage IV breast cancer and did not include women who may have been diagnosed at an earlier stage and then progressed.

## Conclusions

The ER + metastatic breast cancer population is an understudied sub-type of breast cancer. This retrospective analysis found that there is a significant excess clinical and economic burden among women with metastatic ER + breast cancer who were not prescribed a HER2 targeted agent when compared to age, sex, and race matched patients without cancer. Future studies with more precise and recent clinical data paired with cost data are needed in this population to confirm and extend these results.

## Competing interests

KL, HH, VF, and JM are employees of Boston Health Economics, Inc. and were funded by Novartis Pharmaceuticals Corporation for this study. MS and YH are employees the sponsor. The authors have no other financial or non-financial competing interests to disclose.

## Authors’ contributions

KL, MS, and JM were involved in the conception and design of the study, the interpretation of data, and revised the manuscript critically. HH, VF, and YH contributed to the design of the study, the acquisition of data, the analysis and interpretation of data, and drafting the manuscript. All authors have read and approved the final manuscript.

## Pre-publication history

The pre-publication history for this paper can be accessed here:

http://www.biomedcentral.com/1472-6963/14/298/prepub
